# Meta-Analysis of *TNF* 308 G/A Polymorphism and Type 2 Diabetes Mellitus

**DOI:** 10.1371/journal.pone.0018480

**Published:** 2011-04-08

**Authors:** Ren-Nan Feng, Chen Zhao, Chang-Hao Sun, Ying Li

**Affiliations:** Department of Nutrition and Food Hygiene, School of Public Health, Harbin Medical University, Harbin, China; Mayo Clinic College of Medicine, United States of America

## Abstract

**Background and Objectives:**

Many investigations have focused the association between *TNF* 308 G/A polymorphism and risk for type 2 diabetes mellitus (T2DM). However, the sample sizes of most of the studies were small. The aim of this study is to evaluate the precise association between this variant and risk for T2DM in a large-scale meta-analysis.

**Methods:**

All publications were searched on the association between *TNF* 308 G/A polymorphism and T2DM. The key words were as follows: diabetes, tumor necrosis factor and polymorphism/variant/genotype. This meta-analysis was assessed by Review manager 5.0.

**Results:**

There were 18 studies identified. The odd ritos (ORs) and 95% confidence intervals (CI) for GA+AA versus GG genotype of *TNF* 308 G/A polymorphism were 1.03 (0.95–1.12), 1.03 (0.94–1.13) and 1.03 (0.78–1.36) in overall, Caucasian and Asian populations, respectively. The sensitivity analysis further strengthened the validity of this association. No publication bias or heterogeneity was observed in this study.

**Conclusion:**

In summary, there was no significant association detected between the *TNF* 308 G/A polymorphism and risk for T2DM.

## Introduction

The prevalence of diabetes is high, with high rates of morbidity and mortality. World Health Organization (WHO) estimations predicted that by the year 2030, 350 million individuals worldwide would suffer diabetes [1]. Recent decades, chronic inflammation has been found in obesity and diabetes [2]. Tumor necrosis factor alpha (TNF-α) was found to be a crucial component of the pro-inflammatory cytokines, mainly produced by macrophages and adipocytes [3], and can induce insulin resistance in T2DM [4].

As genetic variations in the promoter region may regulate TNF-α production, single nucleotide polymorphisms in the promoter region of the *TNF* gene, such as −238, −308, −857, −1031 in human [5], have been investigated for their roles on gene transcription as well as for their possible relationships with inflammatory related diseases. There were studies focused on *TNF* 308 G/A polymorphism found guanine (G) replaced by adenine (A) in *TNF* 308 position [6], which led to a higher rate of *TNF* gene transcription than that of wild-type in vitro expression studies [7], [8]. For diabetes, our previous study found that *TNF* 308 G/A polymorphism as a risk factor for type 1 diabetes mellitus (T1DM) [9]. However, the association between this variation and risk for T2DM still remained unclear. Since studies were limited to modest sample size and different ethnicity, every single study may be underpowered to achieve a comprehensive and reliable conclusion, and a meta-analysis was required, which has been recently suggested by Boraska *et al.* [10].

In this study, we have therefore conducted a meta-analysis from all eligible studies to confirm whether *TNF* 308 G/A polymorphism would be associated with the risk for T2DM.

## Methods

### Study Selection

We searched several databases (Medline, PubMed and EMBase) through November, 2010 for all publications on the association between TNF 308 G/A polymorphism and T2DM. The key words were as follows: diabetes, tumor necrosis factor and polymorphism or variant or genotype. In addition, we also searched references of retrieved articles. Studies should meet the following criteria: (1) case-control study; (2) only diabetes as outcome, and (3) at least two comparison groups (diabetes vs. control groups) involved in a single study. Exclusive criteria: no report about the genotype frequency, or insufficient information for data extraction. The MOOSE Checklist and the flow chart for the studies were shown as Checklist S1 and Figure S1 in the supporting information.

Finally, we identified 18 studies on the association between *TNF* 308 G/A polymorphism and the risk for T2DM [10]–[27].

### Data Extraction

Two authors extracted data independently and in duplicate, and reached on all items, including: author's last name, journal and year of publication, country of origin, selection and characteristics of diabetes cases and controls, ethnicity of the study population, genotypes (rs1800629) and numbers of cases and controls. The results were compared and disagreements were discussed and resolved with consensus.

### Statistical analysis

The meta-analysis was performed by using Review manager 5.0. We pooled the odds Ratios (ORs) for TNF 308 GA+AA versus GG genotype and further conducted subgroup analyses by ethnicity. Heterogeneity among studies was examined with *I^2^* statistics that was interpreted as the proportion of total variation contributed by between-study variation. If there was no statistical heterogeneity among studies (*I^2^*<50% and *P*>0.05), the ORs and 95% CI would be estimated for each study in a fixed-effects model. Otherwise, a random-effect model should be employed. In sensitivity analysis, relative influence of each study on the pooled estimate was assessed by omitting one study at a time. Funnel plots were used to evaluate publication bias. All *P*-values were two-tailed.

## Results

### Characteristics of the articles in our meta-analysis

The detailed characteristics of the studies included were shown in Table 1. The details for the study searching were shown in Figure S1. There were 18 studies met the inclusion criteria in this meta-analysis of 7,611 T2DM patients and 6,944 controls in Caucasian (n = 9), Asian (n = 6), and other (n = 3) populations, published between 1995 and 2010 [10]–[27].

**Table 1:** Characteristics of publications included in meta-analysis of *TNF* 308 G/A polymorphism and T2DM.

Reference	Country	Patients	Controls	Case/Control (age year)	Case/Control (n)
Bouhaha, 2010	Tunis	T2DM per ADA criteria M/F:84/144	Healthy participants M/F:218/87	43.8/60.6	228/300
Boraska, 2010	UK	T2DM patients	Healthy participants	—	1454/2504
Furta, 2002	Japan	T2DM per ADA criteria M/F:78/54	Healthy participants M/F:111/31	55.6/51.8	132/142
Heijmans, 2002	Netherlands	T2DM patients	Healthy participants	≥85	79/577
Hamann, 1995	German	T2DM patients	Unrelated healthy participants	57.9/56.1	138/57
Ishii, 2000	Japan	T2DM male patients	Healthy male participants	58.76/41.96	71/299
Kim, 2006	Korea	T2DM patients	Healthy participants	—	169/198
Ko, 2003	China	T2DM patients M/F:93/246	Non-diabetic participants, M/F:70/132	38.2/36.8	339/202
Li, 2003	Finland	T2DM patients M/F:173 /222	Healthy participants M/F:129∶155	65.7/55.0	395/284
Lindholm, 2008	Sweden	T2DM per WHO criteria M/F	Non-diabetic participants M/F:107/99	61.2/59.7	2957/206
Liu,2008	China	T2DM patients M/F:132/113	Healthy volenteers M/F:68/54	48.2/46.5	245/122
Morris, 2003	Australia	T2DM patients M/F	Healthy participants M/F	—	91/189
Padovani, 2000	Brazil	T2DM patients	Non-diabetes patients	43/42	21/145
Santos,2006	Chile	T2DM female patients	Healthy female participants	60–69	30/53
Shiau, 2003	China	T2DM patients M/F:142/119	Non-diabetic participants M/F:99/90	58.73/58.16	261/189
Tsiavou, 2004	Greece	T2DM patients M/F:8/24	Healthy participants M/F:44/15	51/44	32/39
Vendrell, 2003	Spain	T2DM per WHO criteria M/F:65/70	Healthy participants M/F:116/91	56.67	135/207
Zeggini, 2005	UK	T2DM patients	Unrelated participants	—	834/1231

M/F: Male/Female; ADA: American Diabetes Association.

### Association between TNF 308 G/A polymorphism and the risk for T2DM

**Figure 1:**
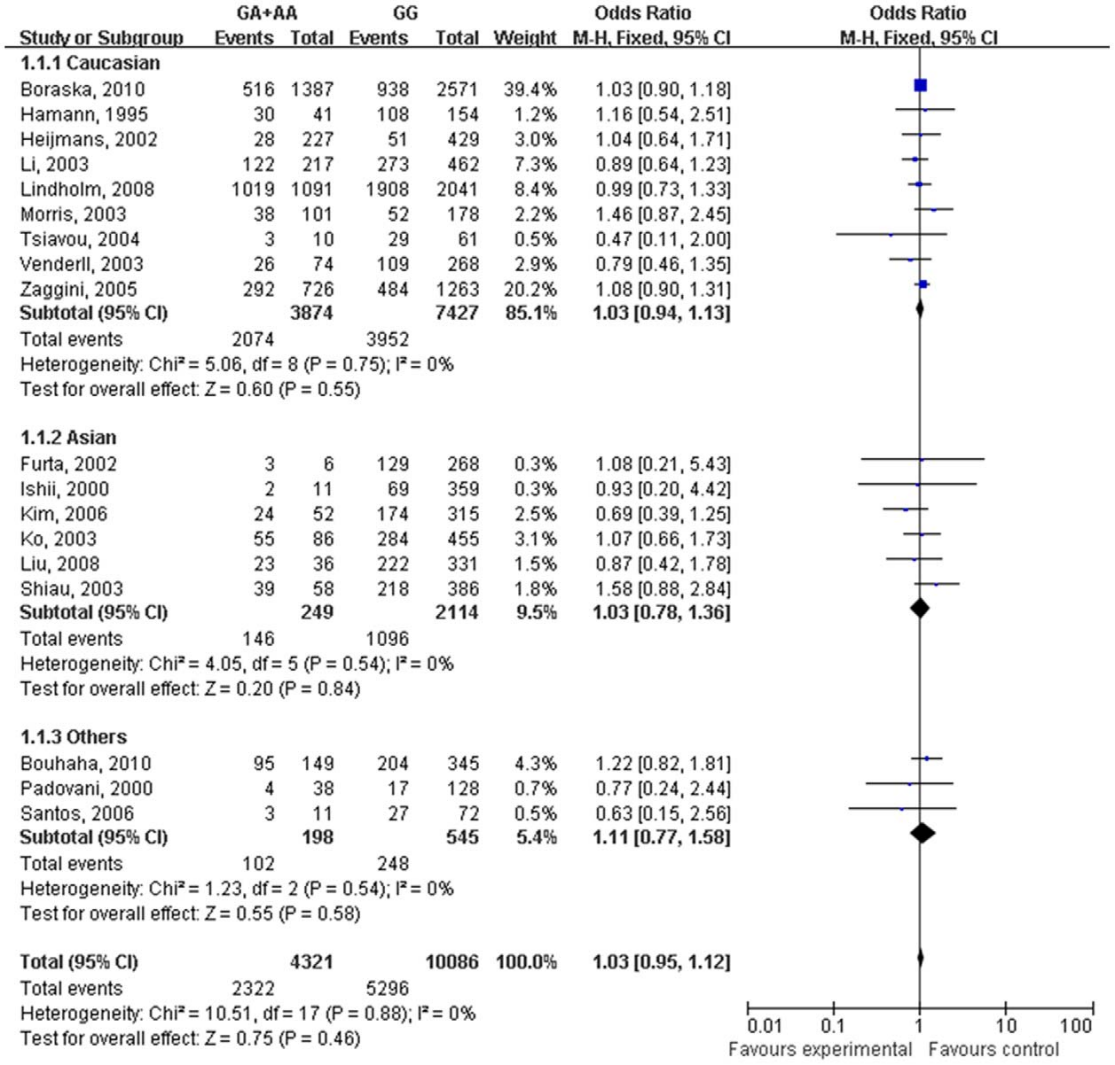
Meta-analysis of the association between *TNF* 308 G/A polymorphism and the risk for type 2 diabetes mellitus.

It has been shown in Figure 1 that the risk for T2DM conferred by *TNF* 308 G/A polymorphism in the overall 18 studies and subgroups did not reach the significant difference. The ORs of GA+AA versus GG for T2DM were 1.03 (0.95–1.12) for the overall, 1.03 (0.94–1.13) for Caucasian and 1.03 (0.78–1.36) for Asian, respectively. There was no evidence of heterogeneity among the overall 18 studies or subgroups (*I^2^* = 0 in overall populations and subgroups).

To further strengthen the confidence for the results, we conducted a sensitivity analysis. This analysis confirmed the stability of the null association between *TNF* 308 G/A polymorphism and T2DM (Table 2). Exclusion of individual studies did not modify the estimates much, with pooled ORs ranging from 1.02 to 1.04.

**Table 2:** Sensitivity analysis with each study omitted in fixed-effects model.

Study omitted	OR	95% CI	*P*
***None***	1.03	0.95–1.12	0.46
***Boraska *** ** *et al.*	1.03	0.93–1.15	0.55
***Hamann *** *et al.*	1.03	0.95–1.12	0.48
***Heijmans *** *et al.*	1.03	0.93–1.13	0.47
***Li *** *et al.*	1.04	0.96–1.14	0.34
***Lindholm *** *et al.*	1.04	0.95–1.13	0.42
***Morris *** *et al.*	1.02	0.94–1.12	0.6
***Tsiavou *** *et al.*	1.04	0.95–1.13	0.42
***Vendrell *** *et al.*	1.04	0.95–1.13	0.37
***Zeggini *** *et al.*	1.02	0.93–1.12	0.68
***Furta *** *et al.*	1.03	0.95–1.12	0.46
***Ishii *** *et al.*	1.03	0.95–1.12	0.45
***Kim *** *et al.*	1.04	0.96–1.14	0.35
***Ko *** *et al.*	1.03	0.95–1.12	0.48
***Liu *** *et al.*	1.04	0.95–1.13	0.43
***Shiau *** *et al.*	1.02	0.94–1.11	0.6
***Bouhaha *** *et al.*	1.02	0.94–1.12	0.58
***Padovani *** *et al.*	1.03	0.95–1.13	0.43
***Santos *** *et al.*	1.03	0.95–1.13	0.43

OR, odds ratio.

**Figure 2:**
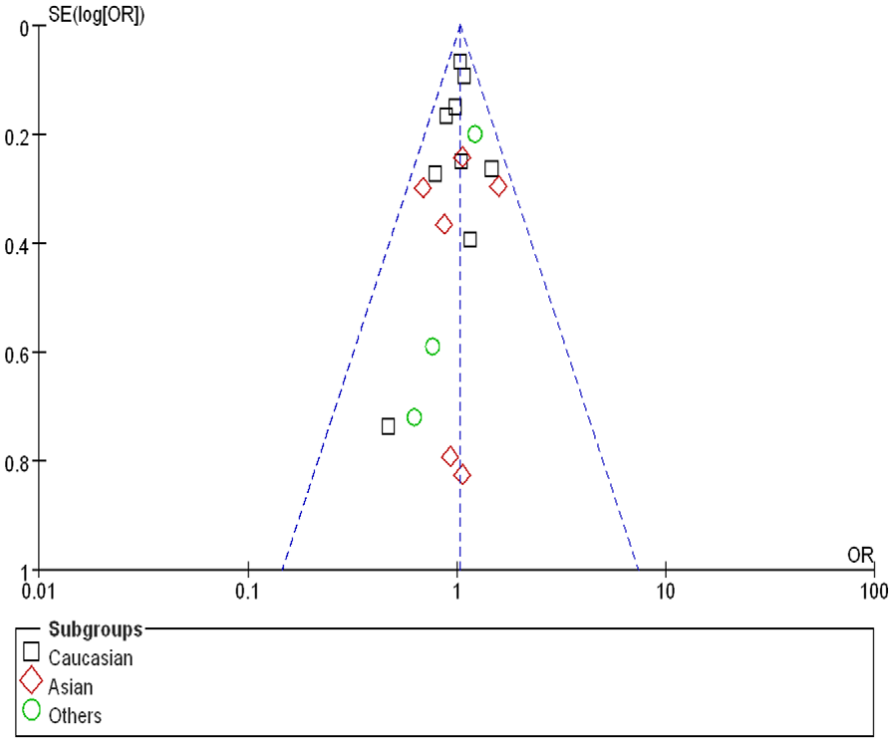
Funnel plots analysis to detect publication bias. Each point represents an independent study for the indicated association.

The shape of the funnel plots was symmetrical, suggesting there was no evidence of publication bias among the studies (Figure 2). Together with the above results, this meta-analysis showed no significant association between *TNF* 308 G/A polymorphism and the risk for T2DM.

## Discussion

Inflammation has been widely known as an important feature of T2DM, with high levels of pro-inflammation cytokines, including IL-1, IL-6 and TNF-α. As TNF-α can impair insulin signal pathways and lead to B-cell destruction, elevated TNF-α is considered playing a central role in the development of T2DM. The *TNF* gene is located within the *HLA* III region in chromosome 6p21, involved in inflammatory responses [28]. Additionally, *TNF* 308 G/A single nucleotide polymorphism (SNP) in the promoter region of *TNF* was found to increase the expression of this pro-inflammatory cytokine in culture cells and positive associated with risk for T1DM [9], [29]. So far, many studies had focused on association between *TNF* 308 G/A polymorphism and T2DM, but the results were still unclear.

In this large-scale meta-analysis, the combined evidence suggested that *TNF* 308 G/A polymorphism did not contribute to the development of T2DM. However, T2DM is a complex disease, and both environmental and genetic factors are involved in the development of T2DM. There are some points should be concerned for the inconsistent results in early reports. Firstly, ethnic differences may attribute to these different results, since the distributions of the *TNF* 308 G/A polymorphism were different between various ethnic populations. For instance, the frequencies of *TNF* 308 G/A polymorphism allele differs from 9% in Chinese population [30], 16% in French and Scandinavian populations [31], [32], 18% in German [33], to 24% in Australians [34]. On the other hand, study design or small sample size or some environmental factors may affect the results. Most of these studies did not consider most of the important environmental factors. It is possible that variation at this locus has modest effects on T2DM, but environmental factors may predominate in the progress of T2DM, and mask the effects of this variation. Specific environmental factors like lifestyle and obesity that have been already well studied in recent decades [35]. Higher free fatty acids are considered as a major risk for T2DM, which can stimulate TNF-α secretion. It is still unknown whether the lifestyle characteristics of different populations influence the association between genotype and T2DM and whether genetic factors influence the ages of onset of T2DM. The unconsidered factors mixed together may cover the role of *TNF* 308 G/A polymorphism. Thus, even if the variation has a causal effect on T2DM, it may take a long time to be observed. Supporting this deduction, Ishii *et al.* reported that FPG in older men was higher and a trend for triglycerides to be higher and HDL cholesterol to be lower in the group with *TNF* 308 G/A polymorphism, but not in the young [14]. Insulin resistance may aggravate gradually in those Japanese subjects together with metabolic changes, such as hyperglycemia and dyslipidemia, on the presence of the *TNF* 308 G/A polymorphism. The effects of the *TNF* 308 G/A polymorphism are so small that in the short period the difference can not be observed. In addition, interaction of the *TNF* gene with other pro- and anti- inflammatory cytokine genes plays an integrated role in destruction of pancreatic beta cells [36]. And cytokines in the circulation interact with each other in the pathogenesis of diabetes. Therefore, it is not surprising that no influence of *TNF* 308 G/A polymorphism was found in susceptibility to T2DM.

However, this variant was reported to be related to some metabolic disorders and T1DM. Furta *et al.* found that TNF-α levels were not significantly different between Japanese T2DM patients with and without this variation [11] and TNF-α levels were affected by visceral fat area, consisting with Morris *et al.* [20]. Moreover, a meta-analysis on relation between *TNF* 308 G/A polymorphism and metabolic syndrome had indicated that individuals carrying TNFA allele had significantly higher fasting insulin level, systolic arterial blood pressure, higher risk of developing obesity and maybe HOMA-IR, but no significant association with BMI. WHR, fasting glucose and plasma leptin levels, which suggested TNFA allele would increase the risk of metabolic syndrome [37]. Also, It was reported that *TNF* 308 G/A polymorphism was more common in T2DM patients with than without macrovascular disease [18]. And our previous study confirmed that a higher frequency of *TNF* 308 A allele conferred a significant risk for T1DM [9]. All the available findings indicated that *TNF* 308 G/A polymorphism might not increase the development of T2DM, but it can enhance the risk for human metabolic disorder and some autoimmunity diseases.

In **c**onclusion, we did not find any evidence of association between *TNF* 308 G/A polymorphism and T2DM in this large-scale meta-analysis. Further prospective research, with larger numbers of participants and fully confounding risk factors considered, such as age, sex, ethnicity and life style, is warrant to examine the possible effects of this variation on T2DM to confirm our conclusion.

## Supporting Information

Checklist S1 (DOC)

Figure S1 The flow chart of the included studies.
(DOC)

## References

[1] Wild S, Roglic G, Green A, Sicree R, King H (2004). Global prevalence of diabetes: estimates for the year 2000 and projections for 2030.. Diabetes Care.

[2] Hotamisligil GS (2006). Inflammation and metabolic disorders.. Nature.

[3] Dahlen E, Dawe K, Ohlsson L, Hedlund G (1998). Dendritic cells and macrophages are the first and major producers of TNF-alpha in pancreatic islets in the nonobese diabetic mouse.. J Immunol.

[4] Hotamisligil GS, Shargill NS, Spiegelman BM (1993). Adipose expression of tumor necrosis factor-alpha: direct role in obesity-linked insulin resistance.. Science.

[5] Feng R, Li Y, Zhao D, Wang C, Niu Y (2009). Lack of association between TNF 238 G/A polymorphism and type 2 diabetes: a meta-analysis.. Acta Diabetol.

[6] Pociot F, Wilson AG, Nerup J, Duff GW (1993). No independent association between a tumor necrosis factor-alpha promotor region polymorphism and insulin-dependent diabetes mellitus.. Eur J Immunol.

[7] Wu X, Zhang WJ, Witt CS, Abraham LJ, Christiansen FT (1992). Haplospecific polymorphism between HLA B and tumor necrosis factor.. Hum Immunol.

[8] Louis E, Franchimont D, Piron A, Gevaert Y, Schaaf-Lafontaine N (1998). Tumour necrosis factor (TNF) gene polymorphism influences TNF-alpha production in lipopolysaccharide (LPS)-stimulated whole blood cell culture in healthy humans.. Clin Exp Immunol.

[9] Feng RN, Li Y, Sun CH (2009). TNF 308 G/A polymorphism and type 1 diabetes: a meta-analysis.. Diabetes Res Clin Pract.

[10] Boraska V, Rayner NW, Groves CJ, Frayling TM, Diakite M (2010). Large-scale association analysis of TNF/LTA gene region polymorphisms in type 2 diabetes.. BMC Med Genet.

[11] Furuta M, Yano Y, Ito K, Gabazza EC, Katsuki A (2002). Relationship of the tumor necrosis factor-alpha −308 A/G promoter polymorphism with insulin sensitivity and abdominal fat distribution in Japanese patients with type 2 diabetes mellitus.. Diabetes Res Clin Pract.

[12] Hamann A, Mantzoros C, Vidal-Puig A, Flier JS (1995). Genetic variability in the TNF-alpha promoter is not associated with type II diabetes mellitus (NIDDM).. Biochem Biophys Res Commun.

[13] Heijmans BT, Westendorp RG, Droog S, Kluft C, Knook DL (2002). Association of the tumour necrosis factor alpha −308G/A polymorphism with the risk of diabetes in an elderly population-based cohort.. Genes Immun.

[14] Ishii T, Hirose H, Saito I, Nishikai K, Maruyama H (2000). Tumor necrosis factor alpha gene G-308A polymorphism, insulin resistance, and fasting plasma glucose in young, older, and diabetic Japanese men.. Metabolism.

[15] Kim HR, Lee MK, Park AJ (2006). [The −308 and −238 Polymorphisms of the TNF-alpha Promoter Gene in Type 2 Diabetes Mellitus.].. Korean J Lab Med.

[16] Ko GT, Lee SC, Pu YB, Ng MC, So WY (2003). Tumour necrosis factor-alpha promoter gene polymorphism at −308 (genotype AA) in Chinese subjects with Type 2 diabetes.. Diabet Med.

[17] Li H, Groop L, Nilsson A, Weng J, Tuomi T (2003). A combination of human leukocyte antigen DQB1*02 and the tumor necrosis factor alpha promoter G308A polymorphism predisposes to an insulin-deficient phenotype in patients with type 2 diabetes.. J Clin Endocrinol Metab.

[18] Lindholm E, Bakhtadze E, Cilio C, Agardh E, Groop L (2008). Association between LTA, TNF and AGER polymorphisms and late diabetic complications.. PLoS ONE.

[19] Liu HL, Lin YG, Wu J, Sun H, Gong ZC (2008). Impact of genetic polymorphisms of leptin and TNF-alpha on rosiglitazone response in Chinese patients with type 2 diabetes.. Eur J Clin Pharmacol.

[20] Morris AM, Heilbronn LK, Noakes M, Kind KL, Clifton PM (2003). −308 Nco I polymorphism of tumour necrosis factor alpha in overweight Caucasians.. Diabetes Res Clin Pract.

[21] Padovani JC, Pazin-Filho A, Simoes MV, Marin-Neto JA, Zago MA (2000). Gene polymorphisms in the TNF locus and the risk of myocardial infarction.. Thromb Res.

[22] Santos MJ, Patino GA, Angel BB, Martinez HJ, Perez BF (2006). [Association between tumor necrosis factor-alpha promoter polymorphisms and type 2 diabetes and obesity in Chilean elderly women].. Rev Med Chil.

[23] Shiau MY, Wu CY, Huang CN, Hu SW, Lin SJ (2003). TNF-alpha polymorphisms and type 2 diabetes mellitus in Taiwanese patients.. Tissue Antigens.

[24] Tsiavou A, Hatziagelaki E, Chaidaroglou A, Manginas A, Koniavitou K (2004). TNF-alpha, TGF-beta1, IL-10, IL-6, gene polymorphisms in latent autoimmune diabetes of adults (LADA) and type 2 diabetes mellitus.. J Clin Immunol.

[25] Vendrell J, Fernandez-Real JM, Gutierrez C, Zamora A, Simon I (2003). A polymorphism in the promoter of the tumor necrosis factor-alpha gene (−308) is associated with coronary heart disease in type 2 diabetic patients.. Atherosclerosis.

[26] Zeggini E, Groves CJ, Parkinson JR, Halford S, Owen KR (2005). Large-scale studies of the association between variation at the TNF/LTA locus and susceptibility to type 2 diabetes.. Diabetologia.

[27] Bouhaha R, Baroudi T, Ennafaa H, Vaillant E, Abid H (2010). Study of TNFalpha −308G/A and IL6 −174G/C polymorphisms in type 2 diabetes and obesity risk in the Tunisian population.. Clin Biochem.

[28] Nishimura M, Obayashi H, Mizuta I, Hara H, Adachi T (2003). TNF, TNF receptor type 1, and allograft inflammatory factor-1 gene polymorphisms in Japanese patients with type 1 diabetes.. Hum Immunol.

[29] Wilson AG, Symons JA, McDowell TL, McDevitt HO, Duff GW (1997). Effects of a polymorphism in the human tumor necrosis factor alpha promoter on transcriptional activation.. Proc Natl Acad Sci U S A.

[30] Lee SC, Pu YB, Thomas GN, Lee ZS, Tomlinson B (2000). Tumor necrosis factor alpha gene G-308A polymorphism in the metabolic syndrome.. Metabolism.

[31] Hoffstedt J, Eriksson P, Hellstrom L, Rossner S, Ryden M (2000). Excessive fat accumulation is associated with the TNF alpha-308 G/A promoter polymorphism in women but not in men.. Diabetologia.

[32] Herrmann SM, Ricard S, Nicaud V, Mallet C, Arveiler D (1998). Polymorphisms of the tumour necrosis factor-alpha gene, coronary heart disease and obesity.. Eur J Clin Invest.

[33] Brand E, Schorr U, Kunz I, Kertmen E, Ringel J (2001). Tumor necrosis factor-alpha–308 G/A polymorphism in obese Caucasians.. Int J Obes Relat Metab Disord.

[34] Dalziel B, Gosby AK, Richman RM, Bryson JM, Caterson ID (2002). Association of the TNF-alpha −308 G/A promoter polymorphism with insulin resistance in obesity.. Obes Res.

[35] Yoon KH, Lee JH, Kim JW, Cho JH, Choi YH (2006). Epidemic obesity and type 2 diabetes in Asia.. Lancet.

[36] Aminkeng F, Van Autreve JE, Koeleman BP, Quartier E, Van Schravendijk C (2007). TNFa microsatellite polymorphism modulates the risk of type 1 diabetes in the Belgian population, independent of HLA-DQ.. Hum Immunol.

[37] Sookoian SC, Gonzalez C, Pirola CJ (2005). Meta-analysis on the G-308A tumor necrosis factor alpha gene variant and phenotypes associated with the metabolic syndrome.. Obes Res.

