# Supplementary material for: Meta-Analysis of TNF 308 G/A Polymorphism and Type 2 Diabetes Mellitus
Source: PLoS One. 2011 Apr 8;6(4):e18480. doi: 10.1371/journal.pone.0018480 (PMC3072982; doi:10.1371/journal.pone.0018480)
Supplement: Checklist S1 — (DOC) [file pone.0018480.s001.doc]

**MOOSE Checklist**

**Meta-analysis of *TNF* 308 G/A polymorphism and type 2 diabetes mellitus**

Ren-Nan Feng·Chen Zhao·Chang-Hao Sun·Ying Li ^*^

Department of Nutrition and Food Hygiene, School of Public Health, Harbin Medical University, Harbin, China

^*^Correspondence: Professor. Ying Li, Department of Nutrition and Food Hygiene, School of Public Health, Harbin Medical University, 157 Baojian Road , Nangang Dstrict, Harbin 150086, P.R. China

Tel: +86 451 87502801; Fax: +86 451 87502885;

E-mail: [liying2002helen@gmail.com](mailto:liying2002helen@gmail.com)

| **Criteria** | | **Brief description of how the criteria were handled in the meta-analysis** |
| --- | --- | --- |
| **Reporting of background should include** | |  |
| √ | Problem definition | Tumor necrosis factor alpha (TNF-α) was found to be a crucial component of the pro-inflammatory cytokines, and play a role in the development of insulin resistance and T2DM. *TNF* 308 G/A polymorphism could lead to a higher rate of *TNF* gene transcription than that of wild-type in vitro expression studies. However, the association between this variation and risk for T2DM still remained unclear. The potential public health impact of *TNF* 308 G/A polymorphism on T2DM remains to be summarized quantitatively. |
| √ | Hypothesis statement | *TNF* 308 G/A polymorphism may increase the risk of T2DM. |
| √ | Description of study outcomes | T2DM |
| √ | Type of exposure or intervention used | GA or AA of *TNF* 308 |
| √ | Type of study designs used | We included case-control studies, cross-sectional studies. |
| √ | Study population | We placed no restriction. |
| **Reporting of search strategy should include** | |  |
| √ | Qualifications of searchers | The credentials of the two investigators RNF and CZ are indicated in the author list. |
| √ | Search strategy, including time period included in the synthesis and keywords | PubMed from 1965 –November 2010  EMBASE from 1974 –November 2010  Medline from 1965 –November 2010  diabetes, tumor necrosis factor and polymorphism or variant or genotype |
| √ | Databases and registries searched | PubMed, Medline and EMBASE |
| √ | Search software used, name and version, including special features | We did not employ a search software. EndNote was used to merge retrieved citations and eliminate duplications |
| √ | Use of hand searching | We hand-searched bibliographies of retrieved papers for additional references, |
| √ | List of citations located and those excluded, including justifications | Details of the literature search process are outlined in the flow chart. The citation list is available upon request |
| √ | Method of addressing articles published in languages other than English | We placed no restrictions on language; local scientists fluent in the original language of the article were contacted for translation |
| √ | Method of handling abstracts and unpublished studies | We had contacted a few colleagues for unpublished studies on the association. |
| √ | Description of any contact with authors | We contacted authors who had conducted multivariate analysis with diabetes as a covariate, but had not reported relative risk for diabetes. |
| **Reporting of methods should include** | |  |
| √ | Description of relevance or appropriateness of studies assembled for assessing the hypothesis to be tested | Detailed inclusion and exclusion criteria were described in the methods section. |
| √ | Rationale for the selection and coding of data | Data extracted from each of the studies were relevant to the population characteristics, study design, exposure, outcome, and possible effect modifiers of the association. |
| √ | Assessment of confounding | No restricted for the analysis. Conducted sensitivity analyses by eliminating each study. |
| √ | Assessment of study quality, including blinding of quality assessors; stratification or regression on possible predictors of study results | The results of sensitivity analyses were very stable. |
| √ | Assessment of heterogeneity | Heterogeneity of the studies were explored within two types of study designs using Cochrane’s Q test of heterogeneity and I^2^ statistic that provides the relative amount of variance of the summary effect due to the between-study heterogeneity. |
| √ | Description of statistical methods in sufficient detail to be replicated | Description of methods of meta-analyses, sensitivity analyses and assessment of publication bias are detailed in the methods. |
| √ | Provision of appropriate tables and graphics | We included the terms used for database search, 1 flow chart,1 summary table, 1 forest plot of all studies,1 funnel plots to examine publish bias, 1 table of sensitivity analyses. |
| **Reporting of results should include** | |  |
| √ | Graph summarizing individual study estimates and overall estimate | Figure 1 |
| √ | Table giving descriptive information for each study included | Table 1 |
| √ | Results of sensitivity testing | Table 2 |
| √ | Indication of statistical uncertainty of findings | 95% confidence intervals were presented with all summary estimates, *P* values and results of sensitivity analyses |
| **Reporting of discussion should include** | |  |
| √ | Quantitative assessment of bias | Sensitivity analyses indicate the null association was very stable. |
| √ | Justification for exclusion | We excluded studies that had used different exposure or outcome assessment for the comparison groups, or no control group. |
| √ | Assessment of quality of included studies | We discussed the results of the sensitivity analyses. |
| **Reporting of conclusions should include** | |  |
| √ | Consideration of alternative explanations for observed results | We discussed that potential unmeasured confounders such as other life style factors and cytokines may have caused residual confounding. |
| √ | Generalization of the conclusions | No association was detected between *TNF* 308 polymorphism and T2DM. We noted the lack of studies in Africa. |
| √ | Guidelines for future research | We recommend future studies on the effect of *TNF* 308 polymorphism and severity of insulin resistance or T2DM. |
| √ | Disclosure of funding source | This research was supported by the National Natural Science Fund of China (No. 30771804 and 30872104) and the Harbin Special Fund for the Technology Innovation Researchers (No. 2009RFLXS203). |
