# Supplementary material for: Meta-Analysis of TNF 308 G/A Polymorphism and Type 2 Diabetes Mellitus
Source: PLoS One. 2011 Apr 8;6(4):e18480. doi: 10.1371/journal.pone.0018480 (PMC3072982; doi:10.1371/journal.pone.0018480)
Supplement: Figure S1 — The flow chart of the included studies. (DOC) [file pone.0018480.s002.doc]

**Flow of Included Studies**

Potentially relevant studies identified and screened for retrieval (n=3398)

Studies excluded, without polymorphism reported (n=3185)

Studies retrieved for more detailed evaluation (n=213)

Potentially appropriate studies to be included in the meta-analysis (n=51)

Studies included in meta-analysis (n=18)

Studies excluded, with no *TNF* 308 genotypes (n=162)

Studies excluded, with no control group or type 1 diabetes as outcome (n=33)
